# Supplementary material for: Computational Chemistry Strategies to Investigate the Antioxidant Activity of Flavonoids—An Overview
Source: Molecules. 2024 Jun 3;29(11):2627. doi: 10.3390/molecules29112627 (PMC11173571; doi:10.3390/molecules29112627)
Supplement: Supplementary file 1 [file molecules-29-02627-s001.zip › molecules-3010931-supplementary.pdf]

Table S1. The flavonoids studied by computational chemistry theory in the past five years.

Flavonoids subclass	Compounds
Flavonoids-glycoside	Abtulin_C [9], Abtulin_D [9], Afzelin [10,11], Ambocin [12]， Apigenin-7-glu [15,19,20], Apigenin-4′-geranyl-8-glucopyranosyl-7-O-α-glucopyranoside [20], Apigenin-4′-pernyl-8-glucopyranosyl-7-O-α-glucopyranoside [20], Brutieridin [23] Cynaroside [37], Didymin [39], Diosmin [21], Hesperetin-5'-O-β-rhamnoglucoside [55,56], Hesperidin [21,37,39,57], Neohesperidin[23,39], Hesperetin-7-glucuronide [55,56], Hibiscetin-3-O-gly [58], Juglanin [11], Kaempferol-3-O-rhamnoside[62][63], Kaempferol-7,4′-dimethylether-3-O-β-d-glucopyranoside [48], Laricetrin-3'-glu [15], Luteolin-7-glu [15], Limocitrin-3-O-(3-hydroxy-3-methylglutarate)-glucoside [39], Myricetin-3-O-rhamnoside [62,63], (Myricetin-3-O-(2″-O-galloyl)-rhamnoside [62,63] Myricitrin [21,60], Naringin [15,21,23,39], Narirutin [37,39], Naritutin-4′-O-glucoside [39], Neoeriocitrin [23], Peripolin [36], Q-3-D-galactoside [73], Q-3-O-rhamnoside [62,63], Q-3-O-β-d-glucoside [63], Querctine-3-O-glucoside [20], Q-3-O-β-d-galactoside [63], Q-3,7-diglu [15], Quercitrin [10], Isoquercitrin [37], Rutin [10,15,61,63], Taxifolin-3-O-α-l-rhamnopyranoside [48], Taxifolin -4′-O-glu [19], Tectorigenin-7-O-[β-D-apiofuranosyl-(1-6)-β-D-glucopyranoside] [12], Vitexin [15]
Flavones /Flavonols	Apigenin [13,15-18,81] Baicalein [16] Caflanone [24] Calycopetrin [25] Chrysin [15,16,35,81] Erysubin F [41], Fisetin [16,17,42,43], Flavones [14,15,29,46,47], 3-Hydroxyflavone [47], 5-Hydroxyflavone [15], 7-Hydroxyflavone [15,21], 3,7-Dihydroxyflavone [47], 3,5-dihydroxyflavone [81], 5-hydroxyflavone [81], 5,7-dihydroxyisoflavone [81], 5-hydroxyisoflavone [81], 3-Hydroxy-7-methoxy-flavone [21], 2'-Hydroxy-flavone [21], 4'-Hydroxy-flavone [21], 4'-Methoxy-flavone [21], 8-Methoxyflavone [15], 5,7,8,5'-Tetramethoxy-3',4'-methylenedioxyflavone [48], 5-Hydroxy-4,7-dimethoxy-6,8-di-C-methylflavone [48], 5,5'',7''-Trihidroksi-3,7-dimetoksi-4'-4'''-O-biflavone [49], Kanzakiflavone [50], 3′-Hydroxyl-Kanzakiflavone [50], 3′-Methoxyl-Kanzakiflavone [50], Flavonols [14]， Galangin [15,43,47,52,78,81], Gossipetin [43], Hibiscetin [58], 3,5,7,3',4',5'-Hexamethoxyflavone [15], Kaempferol[15,16,19,43,47,60,61,78 80 81] Laricytrin [15] Licorice flavonoids [64], Licoflavone [41], Luteolin [16,18,19,81], Morin [15,16,43], Myricetin [15,16,43,46,60,78], Myricetin conformers [65], Nevadensin [18], Norartocarpetin [54], Proxison [63,67], 8-Prenyldaidzein [41], Q [13,15,16,19,26,28,45,47,52,55,56,60,61,63,69-72,78,81], Q-7-methylether [48], Isorhamnetin [16], Robinetin [15,42], Wogonin [16], Xanthomicrol [25], 5-Hydroxyflavone [78], 3,5- dihydroxyflavone [78], Orobol [81]
Isoflavones	Biochanin A [12,21], Eriodyctiol [16] Isoflavones [14,29], Formononetin [21], Genistein [12,16,53,81] 2'-HydroxyGenistein [54], IsoQ [60]

Dihydroflavones/ Dihydroflavonols	Ampelopsin [13], Flavanone [14,15], Citflavanone [28], Fustin [15], Hesperetin [15,21,24,39] Lonchocarpol A [29], Naringenin [13,15,16,21,26,28], DihydroQ [74], Acylated-dihydro Q [74], Silibinin [21], Suaveolensone B [75], Taxifolin [15,21]
Flavans/ Flavanols	Catechin [17,21,26], (+)-Catechin [27,28], 2′-Dimethylamino-(+)-catechin [27], 2′-Trifluoromethyl-(+)-catechin [27], Epicatechin [13,40], Epigallocatechin gallate [40], Flavan [14,44], Flavan-3-ols [14], 3,5,3',4',5'-pentahydroxyflavan-7-gallate [45], 5,3',4',5'-tetrahydroxyflavan-7-gallate [45], 7,4′-O-digalloyltricetiflavan [45], 7,3′-O-di-gallyoltricetiflavan [45]
Chalcones	Broussochalcone [22], Butein [16]， Chalcones [29], 3,4,4'-Trihydroxychalcone [30], 3,4,4'-Trimethydroxychalcone [31], 2',5'-dihydroxy-3,4 -dimethoxychalcone [32], 2',5'-Dihydroxy-3,4-dimethoxychalcone [32], 2',4'-Dihydroxy-4-dimethylaminochalcone [32], 2',5'-Dihydroxy-4-dimethylaminochalcone [32], 2',4'-Dihydroxy-4-dimethychlorochalcone [32], 2′-Hydroxy-3′-aminochalcone [33], 2′-Hydroxy-4′-aminochalcone [33], 2′-Hydroxy-5′-aminochalcone [33], 2′-Hydroxy-3′-cyanochalcone [33], 2′-Hydroxy-4′-cyanochalcone [33], 2′-Hydroxy-5′-cyanochalcone [33], 5-Hydroxy-8-nitro-4’-fluorochalcone [34], 5-Hydroxy-8-nitro-4’-chlorochalcone [34], 5-Hydroxy-8-nitro-4’-bromochalcone [34], 5-Hydroxy-8-nitro-5’- nitrochalcone [34], 5-Hydroxy-8-nitro-4’-methoxychalcone [34], Isobavachalcone [22], Isoliquiritigenin [59], Isoliquiritin [59], Licochalcone [59]， , (E)-3-(2-Chlorophenyl)-1-(2,5-dihydroxyphenyl)prop-2-en-1-one [76], 2-(3-Chlorophenyl)-6-hydroxychroman-4-one[76], (E)-3-(2-Chlorobenzylidene)-2-(2-chlorophenyl)-6-hydroxychroman-4-one [76], (E)-3-(3-Chlorobenzylidene)-2-(3-chlorophenyl)-6-hydroxychroman-4-one[76], (E)-3-(4-Chlorobenzylidene)-2-(4-chlorophenyl)-6-hydroxychroman-4-one [76], Xanthohumol [22], Desmethylxanthohumol [22] Dihydrochalcone [79], 5'-nitrodihydrochalcone [79], 5'-methyldihydrochalcone [79]
Anthocyanins	Anthocyanidins [14] Cyanidin [36] Delphinidin [36,38] Pelargonidin [36], Petunidin [38,66]
Aurones	5-Dihydroxy-3',4'-dimethoxyaurone [32], 6-Dihydroxy-3',4'-dimethoxyaurone [32], 5-Dihydroxy-4'-dimethylaminoaurone [32], 6-Dihydroxy-4'-dimethylaminoaurone [32], 6'-Dihydroxy-4'-dimethychloroaurone [32]
Flavonoids-complex	Chrysin-$B_{12}N_{12}$ complex [35]， Hydroxyflavone-β-Cyclodextrin complex [51], Hydroxyflavone 2-Hydroxypropyl-β-Cyclodextrin complex [51], Procyanidin B1 (PB1) [68], $Cu(PB1)_2$ [68], $Zn(PB1)_2$ [68], Q-β-Cyclodextrin complex [51], Q-2-Hydroxypropyl-β-Cyclodextrin [51], Quercetin-DNA complex [80] Ce(III)-quercetin-phenanthroline complex [82]
Quinones [77]	Q-7-quinone methide, Q-5-quinone methide, Luteolin-ortho-quinone, 3-O-methylQ-ortho-quinone, Isoquercitrin-ortho-quinone

Table S2. The Electronic structure analysis strategies used to investigate the antioxidant of flavonoids in the past five years.

Descriptors	Compounds
The Frontier Molecular Orbitals	Abtulin_C [9], Abtulin_D [9], Afzelin [10,11], Ambocin [12], Ampelopsin [13], Anthocyanidins[14], Apigenin [13,15-18], Apigenin-7-glu [15,19,20], Apigenin-4′-geranyl-8-glucopyranosyl-7-O-α-glucopyranoside [20], Apigenin-4′-pernyl-8-glucopyranosyl-7-O-α-glucopyranoside [20], Baicalein [16], Biochanin A [12,21], Broussochalcone [22], Butein [16], Brutieridin [23], Caflanone [24], Calycopetrin [25], Catechin [17,21,26], (+)-Catechin [27,28], 2′-Dimethylamino-(+)-catechin [27], 2′-Trifluoromethyl-(+)-catechin [27], Chalcones [29], 3,4,4'-Trihydroxychalcone [30], 3,4,4'-Trimethydroxychalcone [31], 2',5'-dihydroxy-3,4 -dimethoxychalcone [32], 2',5'-Dihydroxy-3,4-dimethoxychalcone [32], 2',4'-Dihydroxy-4-dimethylaminochalcone [32], 2',5'-Dihydroxy-4-dimethylaminochalcone [32], 2',4'-Dihydroxy-4-dimethychlorochalcone [32], 2′-Hydroxy-3′-aminochalcone [33], 2′-Hydroxy-4′-aminochalcone [33], 2′-Hydroxy-5′-aminochalcone [33], 2′-Hydroxy-3′-cyanochalcone [33], 2′-Hydroxy-4′-cyanochalcone [33], 2′-Hydroxy-5′-cyanochalcone [33], 5-Hydroxy-8-nitro-4′-fluorochalcone [34], 5-Hydroxy-8-nitro-4′-chlorochalcone [34], 5-Hydroxy-8-nitro-4′-bromochalcone [34], 5-Hydroxy-8-nitro-5′-nitrochalcone [34], 5-Hydroxy-8-nitro-4′-methoxychalcone [34], Isobavachalcone [22], 5-Dihydroxy-3',4'-dimethoxyaurone [32], 6-Dihydroxy-3',4'-dimethoxyaurone [32], 5-Dihydroxy-4'-dimethylaminoaurone [32], 6-Dihydroxy-4'-dimethylaminoaurone [32], 6'-Dihydroxy-4'-dimethychloroaurone [32], Chrysin [15,16,35], Chrysin-$B_{12}N_{12}$ complex [35], Cyanidin [36], Cymaroside [37], Delphinidin [36,38], Didymin [39], Diosmin [21], Epicatechin [13,40], Epigallocatechin gallate [40], Eriodyctiol [16], Erysubin F [41], Fisetin [16,17,42,43], Flavan [14,44], Flavan-3-ols [14], 3,5,3',4',5'-pentahydroxyflavan-7-gallate [45], 5,3',4',5'-tetrahydroxyflavan-7-gallat e[45], 7,4′-O-digalloyltricetiflavan [45], 7,3′-O-di-gallyoltricetiflavan [45], Flavanone [14,15], Citflavanone [28], Flavones [14,15,29,46,47], 3-Hydroxyflavone [47], 5-Hydroxyflavone [15], 7-Hydroxyflavone [15],[21], 3,7-Dihydroxyflavone [47], 3-Hydroxy-7-methoxy-flavone [21], 2'-Hydroxy-flavone [21], 4'-Hydroxy-flavone [21], 4'-Methoxy-flavone [21], 8-Methoxyflavone [15], 5,7,8,5'-Tetramethoxy-3',4'-methylenedioxyflavone [48], 5-Hydroxy-4,7-dimethoxy-6,8-di-C-methylflavone [48], 5,5'',7''-Trihidroksi-3,7-dimetoksi-4'-4'''-O-biflavone [49], Kanzakiflavone [50], 3′-Hydroxyl-Kanzakiflavone [50], 3′-Methoxyl-Kanzakiflavone [50], Hydroxyflavone-β-Cyclodextrin complex [51], Hydroxyflavone 2-Hydroxypropyl-β-Cyclodextrin complex [51], Isoflavones [14,29], Flavonols [14], Fustin [15], Formononetin [21], Galangin [15,43,47,52], Genistein [12,16,53], Gossipetin [43], 2'-HydroxyGenistein [54], Hesperetin [15,21,24,39], Hesperetin-5'-O-β-rhamnoglucoside [55,56], Hesperidin [21,37,39,57], Neohesperidin[23,39], Hesperetin-7-glucuronide [55,56], Hibiscetin [58], Hibiscetin-3-O-gly [58], 3,5,7,3',4',5'-Hexamethoxyflavone [15], Isoliquiritigenin [59], Isoliquiritin [59], Juglanin [11], Kaempferol[15,16,19,43,47,60,61], Kaempferol-3-O-rhamnoside[62,63], Kaempferol-7,4′-dimethylether-3-O-β-d-glucopyranoside [48], Laricytrin [15], Laricetrin-3'-glu [15],

	Licochalcone [59], Licorice flavonoids [64], Licoflavone [41], Lonchocarpol A [29] , Luteolin [16,18,19], Luteolin-7-glu [15], Limocitrin-3-O-(3-hydroxy-3-methylglutarate)-glucoside [39], Melridin [23], Morin [15,16,43], Myricetin [15,16,43,46,60], Myricetin conformers [65], Myricetin-3-O-rhamnoside [62,63], (Myricetin-3-O-(2″-O-galloyl)-rhamnoside [62,63], Myricitrin [21,60], Naringenin[13,15,16,21,26,28], Naringin[15,21,23,39], Narirutin [37,39], Naritutin-4′-O-glucoside [39], Neoeriocitrin [23], Nevadensin [18], Norartocarpetin [54], Pelargonidin [36], Peripolin [36], Petunidin [38,66], Proxison [63,67], 8-Prenyldaidzein [41], Procyanidin B1 (PB1) [68], $Cu(PB1)_2$ [68], $Zn(PB1)_2$ [68], Quercetin [13,15,16,19,26,28,45,47,52,55,56,60,61,63,69-72], Isoquercetin [60], Quercetin-3-D-galactoside [73], Quercetin-3-O-rhamnoside [62,63], Quercetin-β-Cyclodextrin complex [51], Quercetin-2-Hydroxypropyl-β-Cyclodextrin [51], Dihydroquercetin [74], Acylated-dihydroquercetin [74], Quercetin-3-O-β-d-glucoside [63], Querctine-3-O-glucoside [20], Quercetin-3-O-β-d-galactoside [63], Quercetin-3,7-diglu [15], Quercetin-7-methylether [48], Quercitrin [10], Isoquercitrin [37], Isorhamnetin [16], Robinetin [15,42], Rutin [10,15,61,63], Silibinin [21], Suaveolensone B [75], Sylimarine [21],Taxifolin [15,21], Taxifolin-3-O-α-l-rhamnopyranoside [48], Taxifolin -4′-O-glu [19], Tectorigenin-7-O-[β-D-apiofuranosyl-(1-6)-β-D-glucopyranoside] [12], Vitexin [15], Wogonin [16], Xanthomicrol [25], Xanthohumol [22], Desmethylxanthohumol [22], (E)-3-(2-Chlorophenyl)-1-(2,5-dihydroxyphenyl)prop-2-en-1-one [76], 2-(3-Chlorophenyl)-6-hydroxychroman-4-one[76], (E)-3-(2-Chlorobenzylidene)-2-(2-chlorophenyl)-6-hydroxychroman-4-one [76], (E)-3-(3-Chlorobenzylidene)-2-(3-chlorophenyl)-6-hydroxychroman-4-one[76], (E)-3-(4-Chlorobenzylidene)-2-(4-chlorophenyl)-6-hydroxychroman-4-one [76], Reactive intermediates (quinones) [77]: Quercetin-7-quinone methide, Quercetin-5-quinone methide, Luteolin-ortho-quinone, 3-O-methylQuercetin-ortho-quinone, Isoquercitrin-ortho-quinone
Molecular Electrostatic Potential	Abtulin_C [9], Abtulin_D [9], Afzelin [11], Apigenin [17], Apigenin-7-glu [19], Apigenin 7-O-β-d-glucopyranoside [20], Apigenin-4′-geranyl-8-glucopyranosyl-7-O-α-glucopyranoside [20], Apigenin-4′-pernyl-8-glucopyranosyl -7-O-α-glucopyranoside [20], Catechin [17], 2′-hydroxy-3′-aminochalcone [33], 2′-hydroxy-4′-aminochalcone [33], 2′-hydroxy-5′-aminochalcone [33], 2′-hydroxy-3′-cyanochalcone [33], 2′-hydroxy-4′-cyanochalcone [33], 2′-hydroxy-5′-cyanochalcone [33], 5-Hydroxy-8-nitro-4′-fluoro chalcone [34], 5-Hydroxy-8-nitro-4′-chloro chalcone [34], 5-Hydroxy-8-nitro-4′-bromo chalcone [34], 5-Hydroxy-8-nitro-5′- nitro chalcone [34], 5-Hydroxy-8-nitro-4′-methoxy chalcone [34], 3,4,4'-trimethydroxychalcone chalcone[31], Chrysin [35], Chrysin-$B_{12}N_{12}$ complex [35], Epicatechin [40], Epigallocatechin gallate [40], Erysubin F [41], 3,5,3',4',5'-pentahydroxyflavan-7-gallate [45], 5,3',4',5'-tetrahydroxyflavan-7-gallate [45], 7,4′-O-digalloyltricetiflavan [45], 7,3′-O-di-gallyoltricetiflavan [45], 5,7,8,5′-Tetramethoxy-3′,4′-methylenedioxyflavone [48], 5-Hydroxy-4,7-dimethoxy-6,8-di-C-methylflavone [48], 5,5'',7''-Trihidroksi-3,7-dimetoksi-4'-4'''-O-biflavone [49], Fisetin[17], Genistein [53], Hibiscetin [58], Hibiscetin-3-O-gly [58], Juglanin [11], Kaempferol [19,60], Kaempferol-7,4′-dimethylether-3-O-β-d-glucopyranoside [48], Luteolin

	[19], 8-Prenyldaidzein [41], Proxison [67], Quercetin [19,45,60,72], querctine-3-O-glucoside [20], Quercetin-7-methylether [48], Isoquercetin [60], Myricetin [60], Myricetin conformers [65], Myricitrin [60], Suaveolensone B [75], Taxifolin-3-O-α-l-rhamnopyranoside [48], Taxifolin -4′-O-glu [19], (E)-3-(2-Chlorophenyl)-1-(2,5-dihydroxyphenyl)prop-2-en-1-one [76], 2-(3-Chlorophenyl)-6-hydroxychroman-4-one [76], (E)-3-(2-Chlorobenzylidene)-2-(2-chlorophenyl)-6-hydroxychroman-4-one [76], (E)-3-(3-Chlorobenzylidene)-2-(3-chlorophenyl)-6-hydroxychroman-4-one [76], (E)-3-(4-Chlorobenzylidene)-2-(4-chlorophenyl)-6-hydroxychroman-4-on e[76]
Global Descriptive Parameters	Abtulin_C [9], Abtulin_D [9], Afzelin [10,11], Ambocin [12], Apigenin [16], Apigenin-7-glu [19], Baicalein [16], Biochanin A [12], Butein [16], Caflanone [24], Broussochalcone [22], Chrysin [16], Eriodyctiol [16], Fisetin [16,42,43], Flavan [44], Citflavanone [28], Flavone [47], 3-hydroxyflavone [47], 3,7-dihydroxyflavone [47], Galangin [43,47,52], Genistein [12,16], 2′-hydroxygenistein [54], Gossipetin [43], Hesperetin [24], Hibiscetin [58], Hibiscetin-3-O-gly [58], Juglanin [11], Kaempferol [16,19,43,47], Lonchocarpol A [28], Luteolin [16,19], Morin[16,43], Myricetin [16,43], Naringenin [16], Norartocarpetin [54], Petunidin [66], Proxison [67], Quercetin [10,16,19,28,47,52,58,70,71] , Dihydroquercetin [74], Acylated-dihydroquercetin [74], Robinetin [42], Rutin [61], Isorhamnetin [16], Taxifolin -4′-O-glu [19], Tectorigenin-7-O-[β-D-apiofuranosyl-(1-6)-β-D-glucopyranoside][12], Wogonin [16], Suaveolensone B [75], Isobavachalcone [22], Xanthohumol [22], Desmethylxanthohumol [22]
Natural Bond Orbitals	(+)-Catechin [27], 2′-dimethylamino-(+)-Catechin [27], Flavan [44], Flavone [47], 3-hydroxyflavone [47],3,7-dihydroxyflavone [47], Galangin [47], 2′-hydroxygenistein [54], Kaempfero [47], Norartocarpetin [54], Petunidin [66], 2′-trifluoromethyl-Proxison [67], Quercetin [47,[71], Quercetin 3-D-galactoside [73], Suaveolensone B [75]
Natural Transition Orbitals	Galangin [78], Kaempferol [78], Myricetin [78], Quercetin [78], Dihydrochalcone [79], 5'-nitrodihydrochalcone [79], 5'-methyldihydrochalcone [79], 5-Hydroxyflavone [78], 3,5- dihydroxyflavone [78]
Spin Density Distribution	Ambocin [12], Apigenin [17], Biochanin A [12], Catechin [17], 2',5'-dihydroxy-3,4 -dimethoxychalcone [32], 2',5'-dihydroxy-3,4 -dimethoxychalcone [32], 2',4'-dihydroxy-4-dimethylaminochalcone [32], 2',5'-dihydroxy-4-dimethylaminochalcone [32], 2',4'-dihydroxy-4-dimethychlorochalcone [32], Epicatechin [40], Epigallocatechin gallate [40], Fisetin [17,42], Genistein [12], Robinetin [42], Tectorigenin-7-O-[β-D-apiofuranosyl-(1-6)-β-D-glucopyranoside] [12], Suaveolensone B [75]
Dipole Moment/ Polarizability	Ambocin [12], Biochanin A [12], (+)-Catechin [29], 5-Hydroxy-8-nitro-4′-fluoro chalcone [34], 5-Hydroxy-8-nitro-4′- chloro chalcone [34], 5-Hydroxy-8-nitro-4′-bromo chalcone [34], 5-Hydroxy-8-nitro-5′- nitro chalcone [34], 5-Hydroxy-8-nitro-4′-methoxy chalcone [34], Cyanidin [36], Delphinidin [36], Flavone [47], 3-hydroxyflavone [47], 3,7-dihydroxyflavone [47], Galangin [47], Genistein [12], Kaempferol [47],

	Naringenin [29], Pelargonidin [36], Quercetin [29,47], Tectorigenin-7-O-[β-D-apiofuranosyl-(1-6)-β-D-glucopyranoside] [12]
Fuki Function	Ampelopsin [13], Apigenin [13], Epicatechin [13], 2′-hydroxygenistein [54], Naringenin [13], Norartocarpetin [54], Procyanidin B1 [68], Quercetin [13, 80], Quercetin-DNA complex [80], Suaveolensone B [75]
Atomic Charges	Flavone [46], Linebacker [46], Myricetin [46], Proxison [67]
Redox Potentials	Apigenin [81], Chrysin [81], 3,5-dihydroxyflavone [81], 5-hydroxyflavone [81], 5,7-dihydroxyisoflavone [81], 5-hydroxyisoflavone [81] Galangin [81], Genistein [81], Kaempferol [81], Luteolin [81], Quercetin [81], Orobol [81],
Density-of-States	Hibiscetin [58], Hibiscetin-3-O-gly [58], Ce(III)-quercetin-phenanthroline complex [82]
Donator-Acceptor map	Citflavanone [28], Lonchocarpol A [28]
Electron Localization Function	Anthocyanidin [14], Flavan [14], Flavan-3-ol [14], Flavanone [14], Flavonol [14], Flavone [14], Isoflavone [14], Quercetin [13,80], Quercetin-DNA complex [80]
